# Surgical treatment of popliteal cyst: a systematic review and meta-analysis

**DOI:** 10.1186/s13018-016-0356-3

**Published:** 2016-02-15

**Authors:** Xiao-nan Zhou, Bin Li, Jia-shi Wang, Lun-hao Bai

**Affiliations:** Department of Orthopaedics, Shengjing Hospital, China Medical University, 36 Sanhao Street, Heping District, Shenyang, Liaoning 110004 People’s Republic of China

**Keywords:** Popliteal cysts, Surgical intervention, Systematic review, Clinical outcome

## Abstract

**Background:**

This systematic review and meta-analysis of the clinical efficacy of different surgical methods in the therapy of popliteal cysts may provide evidence about effective surgical treatments.

**Methods:**

PubMed, EMBASE, and OVID were searched with the following terms: (popliteal cyst* OR baker’s cyst*) AND (arthroscopic OR excision OR operative OR treat* OR surgery). Inclusion criteria included the following: studies reported the efficacy of different surgical methods in popliteal cyst patients; patients were ≥16 years; and studies must have involved a minimum of 10 patients. Studies were grouped according to the surgical methods, and a meta-analysis was employed to identify the success rate based on the pooled data.

**Results:**

A total of 11 studies were included: The communication between the cyst and the articular cavity was enlarged in 7 studies; this communication was closed in 3 studies; and only intra-articular lesions were managed in 1 study. After the data were pooled, the success rates were 96.7 and 84.6 % in the communication-enlargement group and communication-closure group, respectively. Studies with communication enlargement were subgrouped into the cyst wall resection group and the non-cyst wall resection group, for which the success rates were 98.2 and 94.7 %, respectively.

**Conclusions:**

Based on the current available evidence, at present, any how arthroscopic excision of the cyst wall, arthroscopic management of intra-articular lesions, and enlarging the communication between the cyst and the articular cavity is an ideal strategy for the popliteal cyst. The current literature on the treatment of popliteal cysts is limited to retrospective case series. Future prospective studies with high-quality methodology and uniform scoring system are required to directly compare communication-enlargement surgery and communication-closure surgery and determine the optimal treatment of popliteal cysts. Cyst wall resection may improve the therapeutic efficacy, to draw definitive conclusions, and high-level clinical researches with a large number of patients and long-term follow-up should be initiated.

## Background

Popliteal cysts are a common disease in orthopedics and the most prevalent cystic lesions around the knee joint [1]. They were first recognized by Adama in 1840, and Baker described them in detail in 1877. Accordingly, popliteal cysts are also known as Baker’s cysts [2, 3]. Popliteal cysts most commonly form by distention of the gastrocnemio-semimembranosus bursa, which is located in the medial aspect of the popliteal fossa. The gastrocnemio-semimembranosus bursa is situated between the tendons of the gastrocnemius and semimembranosus muscles and is a normal anatomic finding [4]. A series of studies on the pathogenesis of popliteal cysts revealed the valve structure bridging the cyst and the articular cavity [5, 6]. In adults, popliteal cysts usually occur concomitantly with intra-articular disease, resulting in persistent and excess production of synovial fluid [7, 8]. Sansone et al. found that 94 % of popliteal cysts were associated with a disorder of the knee. The most common disorder was meniscal lesions, followed by anterior cruciate ligament tear and/or chondral lesions. Of the meniscal lesions, 70.2 % were medial meniscal tears, often involving the posterior horn of the medial meniscus [9]. Moreover, the valve structure leads to a one-way flow of the synovial fluid, which finally causes synovial fluid accumulation and subsequent cyst formation. Although asymptomatic popliteal cysts incidentally detected do not require treatment, large cysts may cause popliteal pain or disturbance in the knee range of motion, and they can be the targets of surgical intervention.

Direct excision of the cyst is associated with high rates of recurrence [10, 11]. In 1979, Rauschning and Lindgren reported that the postoperative recurrence rate was as high as 63 % in 40 patients who received open cyst resection via the posterior approach [11]. Several studies have reported frequently associated intra-articular pathologies with the cysts and warned of a high recurrence rate if the intra-articular pathologic condition is not addressed [10, 12, 13]. Lindgren reported on the valvular mechanism of the capsular fold on the posteromedial capsule and continuous unidirectional flow between the posterior joint capsule and gastrocnemius-semimembranosus bursa [14]. If the valvular mechanism or such a communication is not corrected during surgery, the continuous flow of joint fluid will occur, which will lead to a postoperative recurrence. Therefore, surgical treatment includes the following aspects: correction of the intra-articular lesions, closure or enlargement of the communication between the cyst and the articular cavity, and resection of the cyst wall. Arthroscopic correction of the intra-articular disorders aiming at minimizing the risk of recurrent effusions is crucial to prevent relapsing of the cyst. Rupp et al. have completed a study on arthroscopic treatment for intra-articular lesions without any intervention to the cyst [15]. In 5 of the 16 patients, the popliteal cysts had disappeared; 11 cysts had persisted. Some studies reported to close the communication between the cyst and the articular cavity to block synovial fluid flow into the bursae synovialis [16, 17]. Recently, arthroscopic treatment of popliteal cysts by enlarging the communication to abolish the one-way flow has been reported with excellent results in several studies [12, 13, 18–20]. In addition, studies indicated that concomitant resection of the cyst wall which may reduce recurrence rates have yielded very good therapeutic outcomes [21–23].

Although some methods can be used for the surgical treatment of popliteal cysts, none has been confirmed as the best. In this meta-analysis, the authors searched published reports of the outcomes of various surgical interventions for treatment of popliteal cysts and compared the success rates between communication-closure surgery and communication-enlargement surgery. Moreover, on the basis of the heterogeneity of available studies, the influence of cyst wall resection on the success rate was further evaluated. In this study, we sought to determine the effectiveness of currently utilized surgical treatment strategies.

## Methods

### Search strategy

PubMed (1980–July 2015), EMBASE (1980–July 2015), and OVID (1980–July 2015) were searched on 10 July 2015, using the following terms: (popliteal cyst* OR baker’s cyst*) AND (arthroscopic OR excision OR operative OR treat* OR surgery). Inclusion criteria for studies included the following: The popliteal cyst was treated by surgery; postoperative therapeutic efficacy was reported; ≥10 patients in each study; and patients recruited were ≥16 years old. Exclusion criteria included the following: non-English-language articles, reviews, and case reports. All patients had popliteal cyst, and other identical intra-articular diseases were also excluded (such as all patients with rheumatoid cysts of the knee [16]).

Two investigators independently examined the titles of the collected articles and then reviewed the abstract and full text for inclusion in the study. Any discrepancy was resolved by discussion or consultation with the third investigator. In addition, the references of collected articles were also reviewed and could be included if they met the inclusion criteria.

### Methodological quality

The Newcastle–Ottawa scale (NOS) is a simple scale that is easy to use to evaluate the quality of nonrandomized, controlled studies (e.g., case–control studies and cohort studies) [24]. In the NOS, the stars awarded for each quality item served as a quick visual assessment based on the following criteria: the selection of study groups (maximum 4 stars); the comparability of study groups (maximum 2 stars); and the ascertainment of either the exposure or outcome of interest for case–control or cohort studies, respectively (maximum, 3 stars). Thus, the maximum number of stars available for each quality measure was 4, 2, and 3, respectively, and the highest-quality studies could be awarded up to 9 stars. The majority of available studies in orthopedics were case series. Thus, a modified NOS was employed to evaluate the quality of the case series included in our analysis. The original modified NOS was reported by Zengerink et al. to evaluate the quality of case series, and to date, the modified version has been used in systemic reviews [25, 26]. The modified NOS is based on the study design, selection, and outcome, for which the maximum number of stars was 2, 1, and 2, respectively; thus, the highest-quality studies were awarded 5 stars (“Appendix”).

### Data extraction and statistical analysis

Data extraction was performed independently by two investigators. The number of patients included in each study, mean age, sex, mean duration of follow-up, functional score, and success rate were recorded. Any discrepancy was resolved by discussion or by consultation with the third investigator.

The Rauschning and Lindgren (RL) knee score was used to evaluate the outcome and therapeutic efficacy [11]. For the RL clinical score, grade 0 or grade 1 at the last follow-up was employed to define successful therapy. Other clinical scoring tools have not been widely used in clinical practice, but there are similarities between them and the RL clinical score. In the visual analog scale, a score of 1 to 4 was used to define successful therapy [15]. Hughston et al. defined successful therapy as excellent or good according to patients’ objective and subjective statuses [27]. In Rauschning’s study, the clinical scoring tool was similar to the RL scoring tool, and successful therapy was defined as grade 0 or grade 1. According to these criteria, we could calculate the success rate in each study.

Metaprop of R 3.2.1 was employed for the meta-analysis of success rates [28, 29]. A forest plot was used for heterogeneity evaluation, which was determined according to *p* value and *I*^2^ value. A value of *p* > 0.1 or *I*^2^ < 50 % suggested nonevident heterogeneity and a fixed-effects model. On the contrary, if evident heterogeneity was present, a random-effects model was used and the cause of heterogeneity was further explored. Finally, on the basis of heterogeneity and the number of studies included, we investigated the influence of cyst wall resection on the success rate.

## Results

**Fig. 1:**
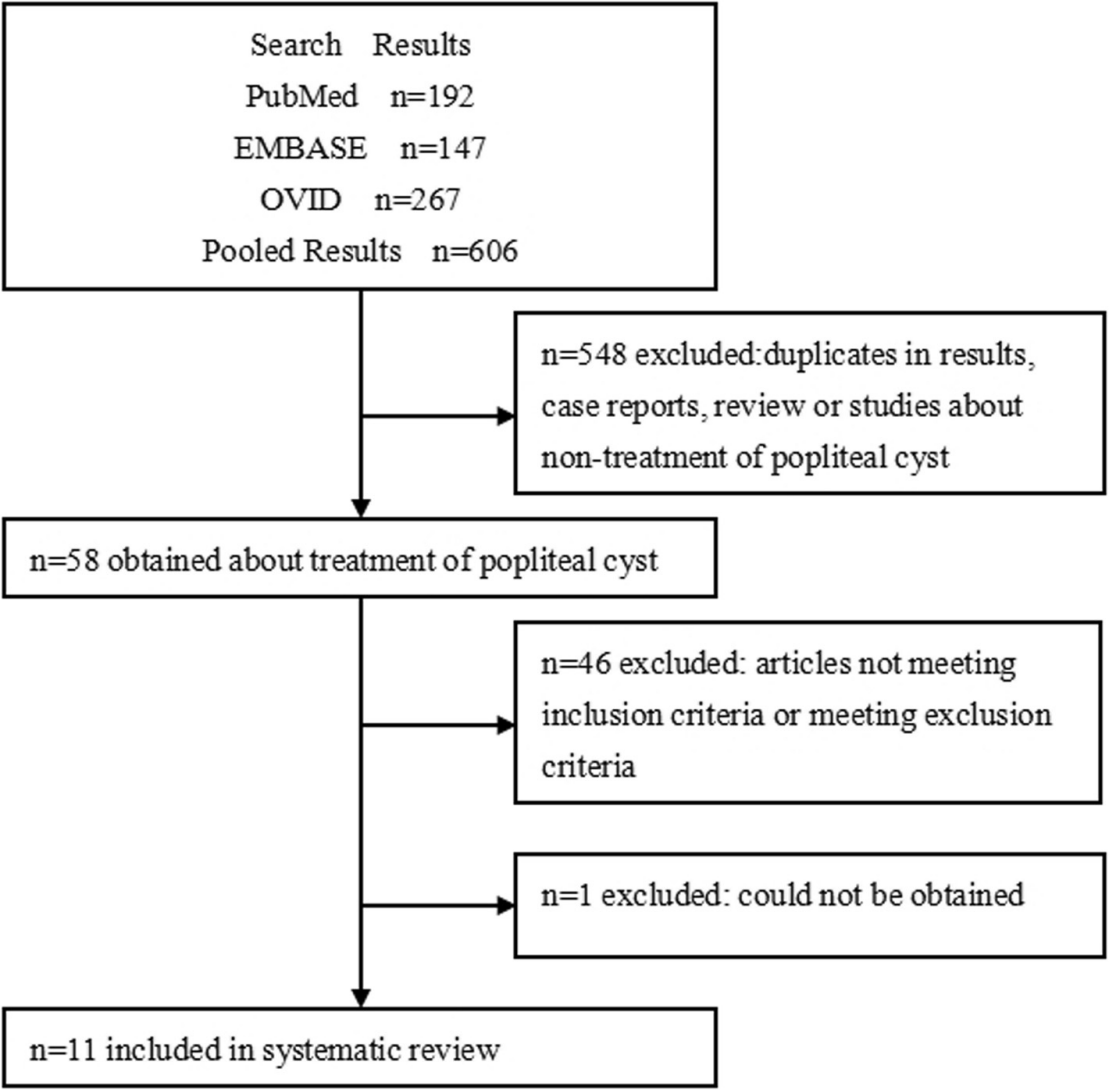
Flow diagram for the selection of publications for systematic review

After searching the databases mentioned above, we identified a total of 606 articles: 192 from PubMed, 147 from EMBASE, and 267 from OVID. Repeated studies, case reports, reviews, and studies unrelated to the therapy of popliteal cysts were excluded, and 58 articles were left. After reviewing the abstract and full text, we screened studies according to the inclusion and exclusion criteria and finally included a total of 11 studies for meta-analysis (Fig. 1) [12, 15, 17, 21, 22, 27, 30–34].

In 7 of the 11 studies, the communication between the cyst and the articular cavity was enlarged by surgery; in 3 studies, the communication was closed; and in 1 study, only intra-articular lesions were managed (Table 1). The success rate was analyzed after pooling of data from studies that involved communication-enlargement surgery and those that involved communication-closure surgery. Among the seven studies with communication enlargement, cyst wall resection was performed in four studies, and the cyst wall was left intact in three studies. In all the seven studies, arthroscopy was employed to treat the popliteal cyst, RL scoring was performed after surgery, and average follow-up time was comparable (Table 2). Thus, we further analyzed the influence of cyst wall resection on the success rate. In the communication-closure group, the number of studies included was small, the tools used for postoperative evaluation were inconsistent, and the measures taken to manage the intra-articular lesions were different; thus, the influence of cyst wall resection on the success rate was not further evaluated even though two studies reported cyst resection. The patients’ characteristics are shown in Tables 2 and 3.

**Table 1:** Studies included in the review and major variables recorded

Authors	Treatment	Newcastle–Ottawa scale	Number of patients	Follow-up (months)	Functional outcomes
Cho [21]	① + ③ + ④	3	111	24	RL: Grade 0, 98 Grade 1, 13
Hua et al. [32]	① + ③ + ④	2	41	18	RL: Grade 0, 40 Grade 1, 1
Ko et al. [34]	① + ③^a^ + ④	3	14	29.7	RL: Grade 0, 14
Ahn et al. [22]	① + ③ + ④^b^	3	31	36.1	RL: Grade 0, 25 Grade 1, 5 Grade 2, 1
Ohishi et al. [30]	① + ③	2	29	22.9	RL: Grade 0, 26 Grade 1, 1 Grade 2, 1 Grade 3, 1
Lie et al. [31]	① + ③	1	10	13	RL: Grade 0, 7 Grade 1, 3
Sansone et al. [12]	① + ③	2	30	32	RL: Grade 0, 19 Grade 1, 10 Grade 2, 1
Calvisi et al. [33]	② + ③	3	22	24	RL: Grade 0, 14 Grade 1, 5 Grade 2, 2 Grade 3, 1
Hughston et al. [27]	② + ③ + ④	1	25	71.6	Excellent: 12 Good: 8, Fair: 3, Poor: 2
Rauschning [17]	② + ③^c^ + ④	2	15	22.3	Grade 0, 14 Clin. Exam. (Recurrent cyst), 1
Rupp et al. [15]	③	4	16	25.2	Visual analog scale Score 1: 2 Score 2: 2 Score 4: 1 Score 9: 2 Score 10: 9

① enlargement of unidirectional valvular slits, ② closed the communication between the articular and the cyst, ③ correctly associated intra-articular disorders, ④ excision of the cyst or removal of the cystic wall

*RL* Rauschning and Lindgren knee score

^a^Simply correctly associated intra-articular disorders

^b^Only 24 patients underwent a direct excisional cystectomy because the other 7 patients presented without fibrous structures

^c^Associated intra-articular disorders were corrected only in a few cases

**Table 2:** Demographic and baseline characteristics of the study patients

	Enlargement	Closure
Number of studies	7	3
Total number of patients	260	61
Mean age	55.0	46.2
Average percent male (%)	31.5	60.7
Average follow-up (months)	25.2	42.3
Total number of functional outcome scores used	1	3

**Table 3:** Patient characteristics stratified by cyst excision

	Excision	Non-excision
Number of studies	4	3
Total number of patients	191	69
Mean age	53.4	59.4
Average percent male (%)	29.3	37.7
Average follow-up (months)	25.1	25.4
Total number of functional outcome scores used	1	1

In addition, 10 of the 11 studies reported concomitant intra-articular lesions in patients with popliteal cysts, and the most common 2 lesions were medial meniscus injury and articular cartilage injury.

### Quality assessment of included studies

Of 11 studies included in this meta-analysis, 10 were case series and 1 was a prospective clinical study [15]. Although there were controls in this prospective study, its goal was to investigate the prevalence of popliteal cysts and the incidence of concomitant intra-articular lesions. This study included no comparator controls vs 20 patients with popliteal cysts. Thus, modified NOS was still used to evaluate its quality.

In terms of study design, 11 studies were awarded a total of 8 of a possible 22 stars. Only one study was prospective, and the remaining designs were retrospective or unknown. In addition, the inclusion and exclusion criteria were described in 7 studies, which accordingly were awarded 7 of 11 possible stars. In terms of selection, 11 studies were awarded 4 of a possible 11 stars because most of the case series were from the same hospital and patients were not recruited consecutively. In terms of outcome, studies were awarded 14 of a possible 22 stars. Ultrasonography or magnetic resonance imaging (MRI) was administered before and after surgery in 7 studies, and their follow-up rate was higher than 95 % in 7 studies. The mean number of stars awarded to these studies was 2.36.

### Meta-analysis

**Fig. 2:**
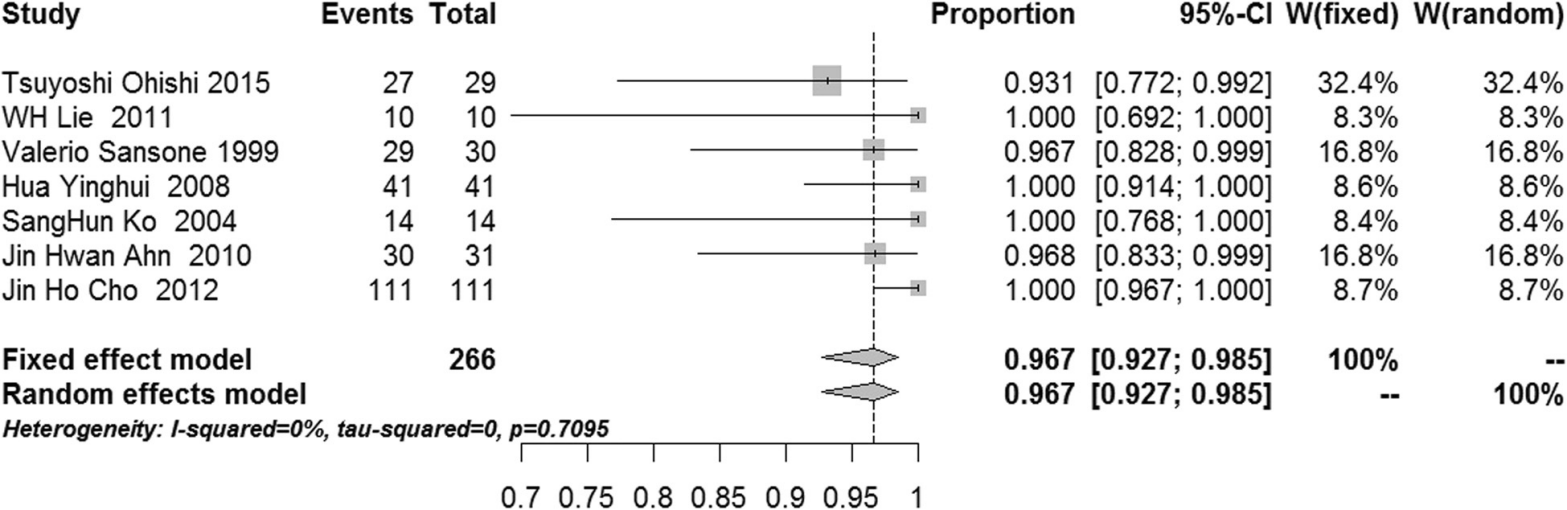
Forest plot of the success rate in the communication-enlargement surgery group

**Fig. 3:**
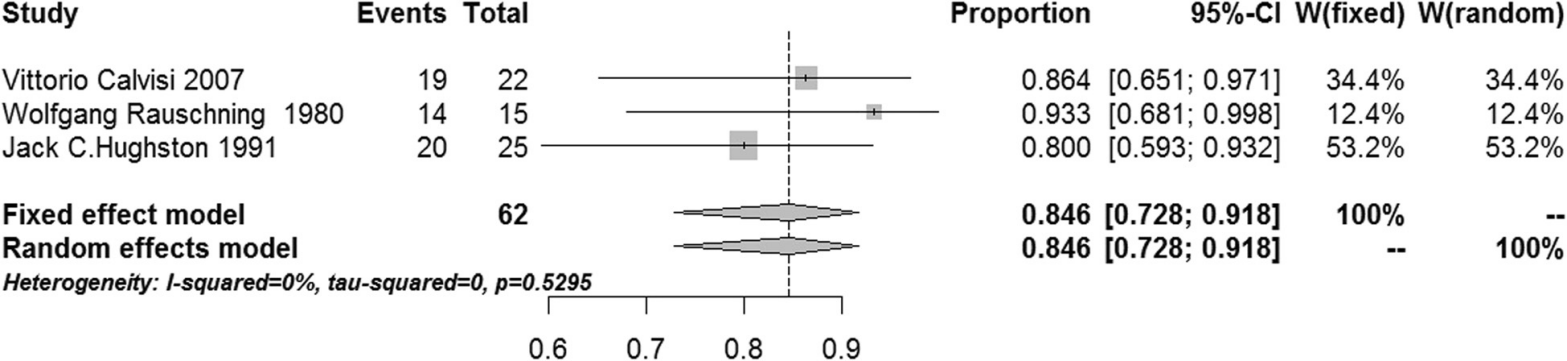
Forest plot of the success rate in the communication-closure surgery group

**Fig. 4:**
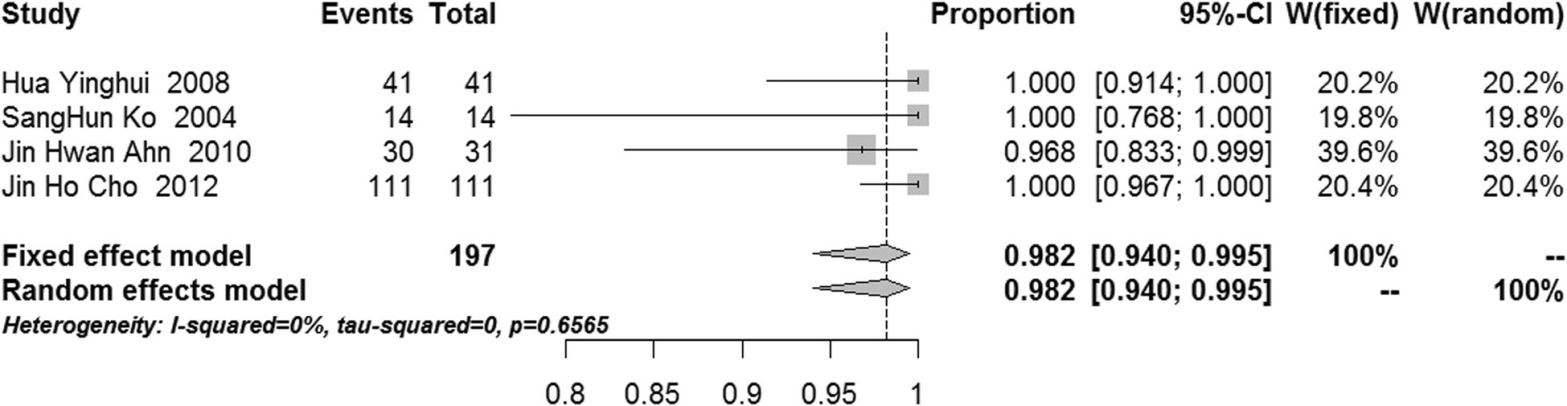
Forest plot of the success rate in the cyst wall resection group

**Fig. 5:**
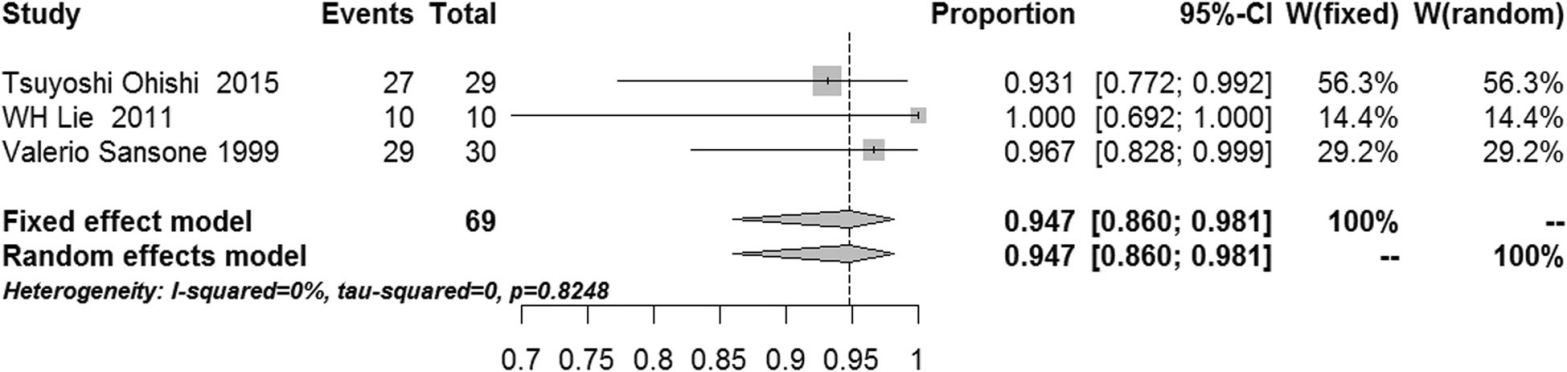
Forest plot of the success rate in the non-cyst wall resection group

After pooling, the success rates of communication-enlargement surgery and communication-closure surgery were 96.7 % (95 % confidence interval (CI) 92.7–98.5 %, *I*^2^ = 0 %, *p* = 0.7095, fixed-effects model) and 84.6 % (95 % CI 72.8–91.8 %, *I*^2^ = 0 %, *p* = 0.5295, fixed-effects model), respectively (Figs. 2 and 3). The meta-analysis showed that the success rates of cyst wall resection group and non-cyst wall resection group were 98.2 % (95 % CI 94.0–99.5 %, *I*^2^ = 0 %, *p* = 0.6565, fixed-effects model) and 94.7 % (95 % CI 86.0–98.1 %, *I*^2^ = 0 %, *p* = 0.8248, fixed-effects model), respectively (Figs. 4 and 5).

## Discussion

This meta-analysis included 11 studies: The communication bridging the cyst and articular cavity was enlarged in 7 studies [12, 21, 22, 30, 32, 34]; the communication was closed in 3 studies [17, 27, 33]; and only intra-articular lesions were managed in 1 study [15]. The most important finding of this study is that the current literature on the treatment of popliteal cysts dose not support one therapeutic method over another due to a lack of high-quality studies. The aim of this study was to pool the data on the treatment of popliteal cysts and determine what is the most effective treatment option for popliteal cysts based on the available literature. The results showed that the success rate was 96.7 % in the communication-enlargement group (no matter if the cyst wall was resected or not). It remains to be seen whether communication-closure surgery is effective, as there is limited literature available on this topic.

Surgical interventions for the treatment of popliteal cysts focus on the management of intra-articular lesions and the communication bridging the cyst and articular cavity, as well as cyst resection. However, open surgical resections of cysts usually result in high recurrence rates [11]. Thus, the therapy for popliteal cysts tends to treat intra-articular lesions and to enlarge or repair the communication between the cyst and the articular cavity. In our meta-analysis, we identified a prospective study in which only intra-articular lesions were managed, and favorable therapeutic efficacy was achieved in only 5 of 16 patients during the follow-up period [15]. Although the number of patients studied was small, this finding suggests that managing intra-articular lesions alone fails to achieve favorable therapeutic efficacy.

While communication enlargement was the most common primary treatment strategy, it was conducted in 7 of the 11 studies in which not only the communication bridging the cyst and articular cavity was enlarged but the intra-articular lesions were also treated [12, 21, 22, 30–32, 34]. After data pooling, we determined that the success rate was 96.7 %. In addition, all the studies employed arthroscopy. The RL score was used after surgery, and statistical analysis showed *I*^2^ = 0 % and *p* = 0.7095. Thus, we speculate that arthroscopic management of intra-articular lesions and enlarging the communication between the cyst and the articular cavity are an effective strategy for the popliteal cyst. Sansone et al. reported the arthroscopic approach to correct the valvular mechanism by opening the connection after removing the posterior horn of the medial meniscus in 1999, and this method also has been modified gradually over time [12]. Ahn et al. found that surgeons could identify the communication between the cyst and the articular cavity using an arthroscope and could enlarge the communication without resection of the posterior horn of the medial meniscus [22]. Recently, Ohishi et al [30] found that only the transverse slit was enlarged in early studies [18, 19, 21, 22, 34], and they suggested that enlarging the vertical slit was also important for the therapy of popliteal cysts. Following the improvement of techniques used to enlarge the communication between the cyst and the articular cavity, the therapeutic efficacy of treatments for popliteal cysts may improve.

In studies of communication-enlargement surgery, cyst wall resection was reported in four studies [21, 22, 32, 34], and patients in three studies did not receive cyst wall resection [12, 30, 31]. Thus, we further investigated the influence of cyst wall resection on the therapeutic outcomes in popliteal cyst patients. The pooled results showed the success rate was 98.2 % after cyst wall resection and 94.7 % in the absence of cyst wall resection. The success rate in the cyst wall resection group was 3.5 % greater compared to that of the non-cyst wall resection group. Although there was good homogeneity in the studies examined, we concluded only that cyst wall resection may benefit patients with popliteal cysts because of the small number of patients recruited. The success rate in the absence of cyst wall resection was also as high as 94.7 %, and thus, additional cyst wall resection may not significantly increase the success rate. However, this result only represents the success rate during the follow-up period (25.2 months), and whether cyst wall resection can improve the long-term efficacy requires confirmation from studies with longer-term follow-up.

In three studies of communication-closure surgery, only several patients received management of intra-articular lesions [17], but this approach was used in all patients in two other studies [27, 33]. After pooling outcomes data, we found that the success rate was as high as 84.6 %, which was improved significantly compared to outcomes following the management of intra-articular lesions alone. In the three studies mentioned above, the original surgery was conducted in an open manner and employed simple suture or using a proximally based flap from the gastrocnemius tendon for additional reinforcement [17, 27], and later arthroscopic all-inside suture was used [33]. Of the 11 studies included in this meta-analysis, arthroscopy was employed in 9 studies reported since 1999. This suggests that arthroscopy has become the treatment of choice for popliteal cysts because of its minimal invasiveness and patients’ rapid postoperative recovery regardless the approaches used. For example, Calvisi et al. reported communication-closure surgery and the management of intra-articular lesions by arthroscopy. Of 22 patients in this study, the success rate was 86.4 %, suggesting favorable efficacy [33]. However, open surgery and arthroscopy were employed for the therapy of popliteal cysts in other available studies, the scoring tools used for postoperative evaluation were different, and a total of only 62 cases were included. Thus, we could not draw any conclusions on the efficacy in treating popliteal cysts. In addition, the success rate which Calvisi et al. reported [33] was comparable to that of the non-cyst wall resection group (94.7 %), but we cannot conclude that the efficacy of communication-enlargement surgery is better than that of communication-closure surgery, because this remains to be validated by additional studies of arthroscopic all-inside suture of symptomatic popliteal cyst.

In 11 studies, investigators reported the incidence of concomitant intra-articular lesions in patients with popliteal cysts, and the results showed that injuries to the medial meniscus and articular cartilage were the most common lesions in the joint. These results remind us to research more effective treatment methods of medial meniscal injuries and articular cartilage injuries may further improve the therapeutic outcomes for treatments of popliteal cysts. In addition, the seven studies that we reviewed also described the reasons for therapeutic failure [12, 15, 17, 22, 27, 30, 33]. Rupp et al. reported that postoperative cysts persisted in patients with higher-level articular lesions (grade 3 or grade 4), but cysts disappeared in patients with lower-level articular lesions [15]. In another 6 studies, therapeutic failure was observed in 13 patients, of whom 5 had knee arthritis, 3 had malacia of the posterior tibial plateau, and 1 had severe chondral defects [12, 17, 22, 27, 30, 33]. This not only confirms the importance of managing intra-articular lesions during surgery but also indicates that the available methods used to treat severe arthritis and cartilage defects are limited.

There were still limitations in this systemic review and meta-analysis: (1) Non-English articles were excluded from this analysis. (2) Included studies were case series with low evidence levels; modified NOS was employed for the evaluation of study quality, and low scores were indicated for these studies. Future high-quality studies are required to confirm the efficacy of surgical interventions to treat popliteal cysts. (3) Only three studies included communication-closure surgery, and only one study reported communication-closure surgery and concomitant management of intra-articular lesions by arthroscopy. Patients who received arthroscopic communication-closure surgery were not further subgrouped. Due to the lack of amount and homogeneity of the literature, no conclusions can be drawn. More studies are needed to investigate the efficacy of communication-closure surgery in patients with popliteal cysts.

## Conclusions

Based on the current available evidence, at present, any how arthroscopic excision of the cyst wall, arthroscopic management of intra-articular lesions, and enlarging the communication between the cyst and the articular cavity is an ideal strategy for the popliteal cyst. The pooled success rates for communication-enlargement surgery and communication-closure surgery were 96.7 and 84.6 %, respectively; the pooled results showed the success rate was 98.2 % after cyst wall resection and 94.7 % in the absence of cyst wall resection. The current literature on the treatment of popliteal cysts is limited to retrospective case series, so the results from this meta-analysis are not available to support one treatment strategy over another. Future prospective studies with high-quality methodology and uniform scoring system are required to directly compare communication-enlargement surgery and communication-closure surgery and determine the optimal treatment of popliteal cysts. Cyst wall resection may improve the therapeutic efficacy, to draw definitive conclusions, and high-level clinical researches with a large number of patients and long-term follow-up should be initiated.

## Appendix

### Newcastle–Ottawa quality assessment scale

Adjusted for case series by Zengerink et al. [25].

Study design

1. Type of study
  - Prospective
  - Retrospective
  - Other
  - Not described
2. Set-up
  - According to protocol
  - Without protocol
  - No protocol described

Selection

1. Representatives of included patients
  - Truly representative of the average community-dwelling chronic syndesmosis patient
  - Somewhat representative of the average community-dwelling chronic syndesmosis patient
  - Group of patients selected by the surgeon
  - No description of the patient’s provenance

Outcome

1. Assessment of outcome
  - Independent blind assessment
  - Record linkage
  - Self-report
  - No description
2. Adequacy of follow-up
  - Complete follow-up—all subjects accounted for
  - Subjects lost to follow-up are unlikely to introduce bias—e.g., a small number lost (<5 %)
  - Follow-up rate of <95 % and no description of those lost to follow-up
  - No statement

## Abbreviations

NOS: Newcastle–Ottawa scale
RL: Rauschning and Lindgren knee score
MRI: magnetic resonance imaging
